# “A loving man has a very huge responsibility”: A mixed methods study of Malawian men’s knowledge and beliefs about cervical cancer

**DOI:** 10.1186/s12889-020-09552-1

**Published:** 2020-10-02

**Authors:** Samuel Lewis, Corrina Moucheraud, Devon Schechinger, Misheck Mphande, Ben Allan Banda, Hitler Sigauke, Paul Kawale, Kathryn Dovel, Risa M. Hoffman

**Affiliations:** 1 University of California Los Angeles David Geffen School of Medicine, Los Angeles, CA USA; 2 University of California Los Angeles Fielding School of Public Health, Los Angeles, CA USA; 3 University of California Los Angeles Meyer and Renee Luskin School of Public Affairs, Los Angeles, CA USA; 4 Partners in Hope Medical Center, Lilongwe, Malawi; 5 African Institute for Development Policy, Lilongwe, Malawi

**Keywords:** Cervical cancer, Screening, Men, Mixed methods, Qualitative, HIV

## Abstract

**Background:**

In Malawi, numerous barriers may prevent women from accessing cervical cancer screening services — including social factors such as male partner involvement. We conducted surveys that included open- and closed-ended questions with married Malawian men to evaluate their knowledge and beliefs about cervical cancer.

**Methods:**

HIV-positive adult (≥18 years) men (married or in a stable relationship) were recruited from an antiretroviral therapy clinic in Lilongwe, Malawi. Men were asked a series of survey questions to assess their knowledge about cervical cancer, experience with cervical cancer, their female partner’s screening history, and their beliefs about gender norms and household decision-making. Following the survey, participants responded to a set of open-ended interview questions about cervical cancer screening, and men’s role in prevention.

**Results:**

One hundred-twenty men were enrolled with average age 44 years and 55% having completed secondary school or higher education. Despite only moderate knowledge about cervical cancer and screening (average assessment score of 62% correct), all men expressed support of cervical cancer screening, and most (86%) believed they should be involved in their female partner’s decision to be screened. Over half (61%) of men said their female partner had previously been screened for cervical cancer, and this was positively correlated with the male respondent having more progressive gender norms around sexual practices. Some men expressed concerns about the screening process, namely the propriety of vaginal exams when performed by male clinicians, and whether the procedure was painful.

**Conclusions:**

Male partners in Malawi want to be involved in decisions about cervical cancer screening, but have limited knowledge about screening, and hold rigid beliefs about gender norms that may affect their support for screening. Messaging campaigns addressing men’s concerns may be instrumental in improving women’s adoption of cervical cancer screening services in Malawi and similar settings.

## Background

Cervical cancer is a major cause of death and disability in low- and middle-income countries [1, 2], despite very effective prevention options [3, 4], including screening approaches that remove abnormal tissue and prevent progression to cancer [5–7]. Although screening has reduced the burden of cervical cancer in wealthy countries, such gains have not yet been achieved in lower-resource settings [8]. Accordingly, the World Health Organization has announced a “global call to action” for cervical cancer elimination worldwide, which includes scaling up coverage of screening [9]. However it is estimated that only 19% of women in low-income countries have been screened for cervical cancer in their lifetime, versus over 60% in high-income countries [10]. Screening coverage must be increased in order to reduce the global inequity of cervical cancer burden and improve outcomes for women in low-income settings [6, 11, 12].

The literature on determinants of screening uptake in lower-resource settings has identified important factors including women’s cervical cancer knowledge, perceived efficacy of screening, and beliefs about disease severity and susceptibility [13–18]. Women have also reported that their male partners influence cervical cancer screening decisions [13, 18–24], and the World Health Organization recommends male-targeted outreach as a way to increase cervical cancer screening uptake [25] – however very little is known about men’s perspectives on cervical cancer and screening approaches [26]. The limited literature on this topic indicates that men are not very knowledgeable about cervical cancer (including risk factors, symptoms and ways of screening or treating), and that despite general support for the concept of cancer prevention, some men have hesitations about their wives undergoing screening [27–38]. Men have specifically reported discomfort with male doctors performing exams, concerns about fertility-related complications, and concerns relating to care access (particularly travel time and cost) [29, 34, 37, 38]. However, it is unknown if and how men’s cervical cancer knowledge and beliefs affect their partner’s screening behavior.

Malawi has the greatest burden of cervical cancer worldwide [1], and screening coverage remains suboptimal [39]. Previous studies have identified numerous barriers to women’s uptake of screening in Malawi, but partner involvement/support has not been explored, despite its importance in other women’s health services in Malawi [40, 41] and elsewhere [42–45]. Moreover, there is a lack of evidence on the relationship between men’s characteristics, including views on cervical cancer and gender attitudes, with partner screening behavior. Therefore, we sought to understand Malawian men’s knowledge, beliefs, experiences, and perceptions of cervical cancer and the screening process, as well as the association of these factors with partner screening. We performed our study among HIV-positive men, given their female partners are either at-risk of HIV infection or HIV-positive, and HIV has been strongly associated with cervical cancer [46, 47]. The study was conducted among HIV-positive men who were clients at an HIV treatment program with active cervical cancer screening services, allowing us to explore cervical cancer perceptions among men who have some level of cervical cancer knowledge. Findings can inform additional messaging that may be required to gain men’s support for cervical cancer screening programs.

The objective of this study was to answer the following questions among men with some level of exposure to cervical cancer screening programs in Malawi: (1) What do HIV-positive Malawian men know and believe about cervical cancer? (2) In this population, is there an association between a man’s partner having ever been screened for cervical cancer, and his knowledge, beliefs, gender attitudes, and household decision-making/−sharing? To address these research questions, we conducted a mixed methods study among adult men in married or long-term relationships at an HIV treatment facility in Lilongwe, Malawi.

## Methods

### Study setting and participant recruitment

Surveys were conducted at a large, free antiretroviral therapy (ART) urban clinic in Lilongwe Malawi, from June–July 2019. Participants were selected through convenience sampling: adult males waiting in the queue for HIV services were randomly approached by the survey administrator to introduce the study and determine eligibility. Men who were ≥ 18 years of age, had a wife or long-term partner (we use “female partner” to encompass both these groups), and had previously heard of cervical cancer were eligible to participate. After obtaining oral informed consent, a male Malawian research assistant with qualitative and quantitative research experience administered the mixed-methods survey in the local language in a private setting at the clinic. All respondents were given a refreshment as a small token of appreciation for participating in the survey.

### Instrument

A mixed-methods survey tool was developed incorporating previously-validated modules from other studies and an interview guide developed for the purposes of this study (Additional files 1 and 2).

The quantitative survey consisted of closed-ended questions on respondent and partner demographics; cervical cancer knowledge, perceptions, and experiences; gender beliefs; and household decision-making (Additional File 1). Knowledge of cervical cancer risk factors and services was assessed through a set of true/false questions adapted from Rosser et al., 2014 [35], and household decision-making was evaluated using Demographic and Health Survey items [48]. Gender beliefs were ascertained through the use of a modified Gender Equitable Men (GEM) scale, a validated tool to assess support for gender equitable norms that has been used in over 20 countries [49–51]. Men who reported having multiple sexual partners or wives were asked to answer all questions about their primary partner only.

The qualitative interview guide included open-ended questions about the respondent’s understanding of cervical cancer and cervical cancer services, and their opinions about the role of men in the prevention of cervical cancer (Additional File 2). All respondents were read a brief description of how screening (using visual inspection with acetic acid, VIA) is conducted and asked about their comfort with the process. Men whose partners had previously been screened were asked questions about their knowledge of their partner’s screening experience.

The final instrument was translated into Chichewa (local language in Malawi) and pilot tested with three respondents before beginning data collection. Interviews lasted between 30 and 45 min. Interview responses were audio recorded with respondents’ permission.

### Data analysis

#### Quantitative

Descriptive statistics were performed to summarize demographic variables and all information about cervical cancer knowledge and awareness, gender beliefs, and household decision-making. A knowledge score was calculated based on responses to true/false statements about cervical cancer disease and services (maximum = 16 points, indicating higher knowledge). A GEM score was calculated from the level of agreement with 8 statements about gender norms (maximum = 16 points, indicating more support for gender equity). Sub-domain scores were also calculated within GEM based on questions about sexual relationships and violence. Bivariate analyses compared GEM and knowledge scores based on reported screening history of the respondent’s partner, using appropriate statistical tests (Pearson’s chi-squared test for categorical variables, two independent samples t-test for continuous variables) and a significance level of *p* < 0.05. Multivariate logistic regression assessed the association between GEM and knowledge scores with partner screening status. Covariate selection was determined through the use of a Directed Acyclic Graph (DAG) for each model (Additional file 3). All analyses were conducted using Stata v16 software (StataCorp 2019).

#### Qualitative

Interviews were translated from Chichewa and transcribed into English. A codebook was developed based on key concepts from the interview guide, and emergent codes were added based on reviewing a subset of 8 transcripts. Coding was performed by three authors (CM, SL, DS), after a process of comparing coding decisions on a common set of 5 transcripts, using NVivo software (QSR International, v12). Themes and variations within codes (by respondent age, knowledge of cervical cancer, and GEM score) were examined inductively across respondents. All quotes are presented with the respondent’s age range and reported partner screening status.

### Ethical review

The study protocol was approved by the Institutional Review Board at the University of California, Los Angeles, and the National Health Sciences Research Committee in Malawi.

## Results

Of 165 men we approached for potential participation, 125 were eligible for inclusion and willing to participate (see Additional file 4 for list of reasons men were screened out; the most common reason for nonparticipation was being unmarried or not having a long-term partner [*n* = 23], followed by never having heard of cervical cancer [*n* = 12] and refusing to participate in the study [*n* = 5]). Five participants were unaware of their partner’s screening status and were excluded from all analyses, as they could not be combined with either group for main comparisons and the small number of men in this group prohibited a subgroup analysis of those unaware of screening status.

Among the 120 respondents who were aware of their partner’s screening status, 73 (61%) reported having a primary partner who had previously screened for cervical cancer, and 47 (39%) reported having an unscreened primary partner (Table 1). Approximately one-fifth of all respondents (*n* = 25, 21%) reported having multiple sexual partners in the past 12 months and 8% (*n* = 10) reported currently having multiple wives. The mean age of respondents with a screened partner was 47 years, versus 42 years among men with an unscreened partner. Primary female partners who were reported to be screened were significantly older (mean age 40 years) than unscreened primary female partners (mean age: 34 years, *p* < 0.01). A higher percentage of men with screened partners were HIV-positive (*n* = 26, 55%), although this was not a significant difference (*p* = 0.07). These differences persisted in age-stratified models, but were more prominent among men under the age of 45 (data not shown).

**Table 1:** Respondent characteristics by partner screening status

	Total^**†**^ ***n*** = 120		Partner screening status			
			Not previously screened ***n*** = 47		Previously screened ***n*** = 73	
**Respondent age (years), mean (range)**	44	(23–71)	42	(23–71)	47	(34–66)
**Respondent level of educational attainment,** ***n*** **%**						
Primary 4 or less	12	10%	7	15%	5	7%
Primary 5–8	42	35%	17	36%	25	34%
Secondary	48	40%	18	38%	30	41%
Beyond secondary	18	15%	5	11%	13	18%
**Respondent occupation,** ***n*** **%**						
Wage employment excluding casual work	53	44%	16	34%	37	51%
Household or self-run business	60	50%	28	60%	32	44%
Casual work	7	6%	3	6%	4	5%
**Respondent financial status past year,** ***n*** **%**						
Income was sufficient and I saved	44	37%	18	38%	26	36%
Only just met expenses	57	48%	23	49%	34	47%
Income insufficient so I used savings or borrowed	19	16%	6	13%	13	18%
**Respondent partnership status,** ***n*** **%**						
One wife	85	71%	32	68%	53	73%
One wife, multiple sexual partners	25	21%	10	21%	15	21%
Multiple wives	10	8%	5	11%	5	7%
**Primary partner age (years), mean (range)**	38	(20–65)	34	(20–65)	40	(23–59)
**Primary partner reported HIV status,** ***n*** **%**						
Positive	78	65%	26	55%	52	71%
Negative or unknown	42	35%	21	45%	21	29%

†Five respondents were unaware of their partner’s screening status and were dropped from all subsequent analyses

### Experiences and knowledge of cervical cancer

Overall, 21% of men (*n* = 25) reported knowing someone who had died from cervical cancer and 10% (*n* = 12) knew someone who had survived cervical cancer (Appendix 1). Approximately 61% of all respondents (*n* = 73) felt that cervical cancer is more dangerous than HIV, and nearly 90% (*n* = 106) agreed that their primary partner was at risk of developing cervical cancer throughout her life. A higher proportion of men with previously-screened partners knew someone who had died or survived cervical cancer, agreed that cervical cancer is more dangerous than HIV, and expressed that their partner was at risk, compared to men whose partners had never been screened, although these were not significant associations.

**Fig. 1:**
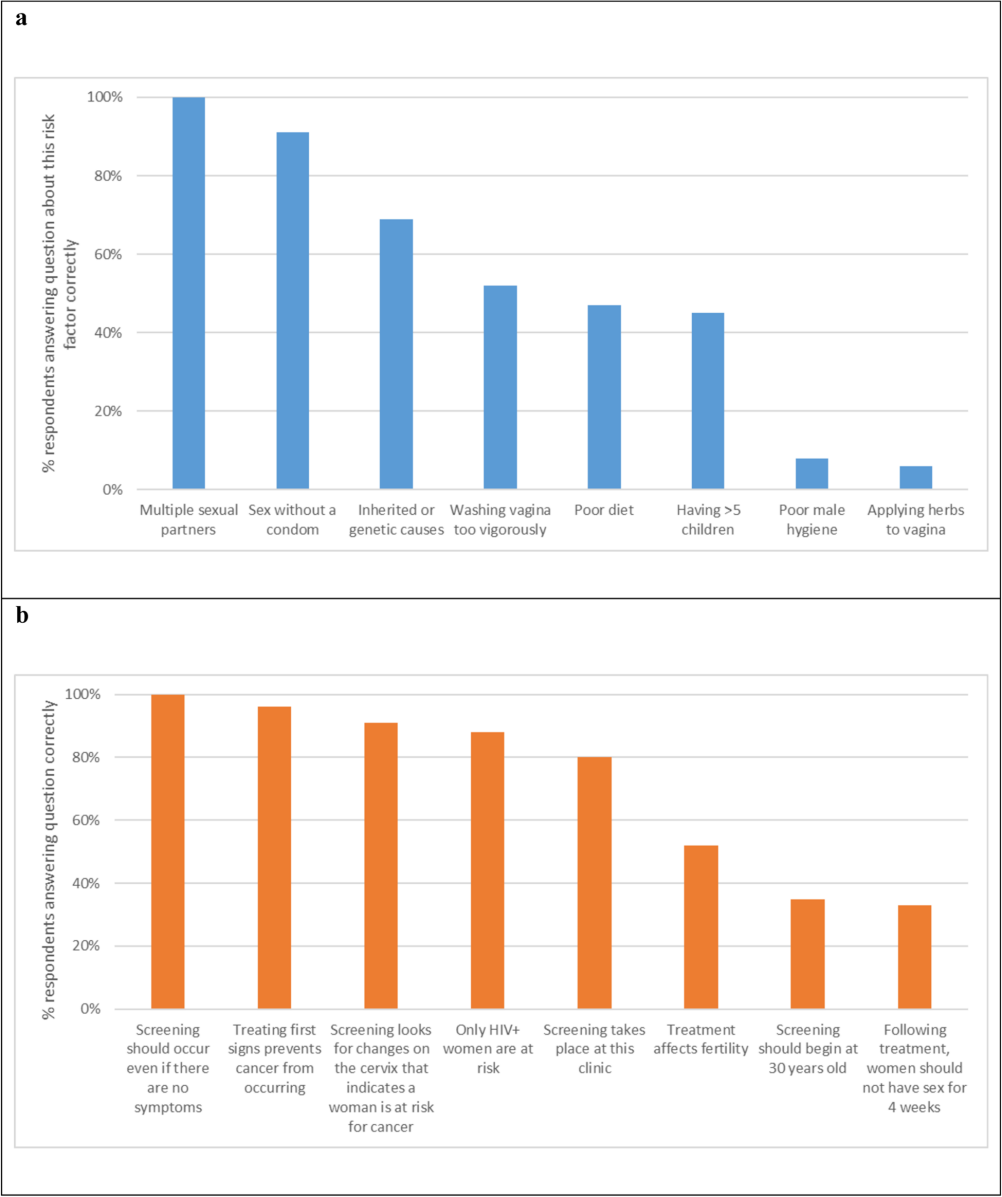
Correct response to knowledge questions about cervical cancer risk factors (**a**) and screening and treatment (**b**)

On average, men responded correctly to 4.2 out of 8 questions about cervical cancer risk factors (52% correct response rate) (Appendix 2) and there was no significant difference by partner screening status (data not shown). Although all respondents answered correctly to certain items (having multiple sexual partners is a risk factor for cervical cancer), other questions were rarely answered correctly (e.g., only 6% of men knew that applying herbs to the vagina is not a risk factor for cervical cancer) (Fig. 1a). There was no significant association between having a screened partner and knowledge of any individual risk factor.

During qualitative interviews, many men spoke about cervical cancer risk factors relating to sexual behavior. The correlation between multiple sexual partners and cervical cancer risk was often mentioned, especially by relatively younger men whose partners had been screened; and younger men with higher GEM and knowledge scores commonly mentioned unprotected sex (both with one’s primary sexual partner, and with other partners) as a risk factor.

*“A man should be faithful to his wife and avoid promiscuity. A woman also has the same responsibility.”* (40–44 years old, partner screened).

*“[Wife and husband] should have protected sex so that they do not get any sexually transmitted diseases from each other.”* (40–44 years old, partner screened).

Respondents expressed that both men and women should maintain genital cleanliness in order to prevent cervical cancer. This response was more common among men whose partners had not been screened. Circumcision was seen as a way to be more hygienic.

*“It is said that sometimes uncleanliness can cause the cervical cancer, so the circumcision brings cleanliness that will also help in the prevention.”* (35–39 years old, partner not screened).

### Knowledge and beliefs about screening

Men responded correctly to an average of 5.8 of 8 questions about screening and treatment services (correct response rate of 72%) (Appendix 2); although men with screened partners had a slightly higher score (74%, versus 69% for men with unscreened partners) this was not a statistically significant difference. Correct responses to individual items ranged from 33% (following treatment, women should not have sex for 4 weeks) to 100% (screening should take place even if there are no symptoms) (Fig. 1b). The only questions with responses significantly associated with whether a man’s partner had been screened were the availability of screening at the study site (OR 2.58, 95% CI: 1.11–5.98), and the need for abstinence following treatment (OR 4.25, 95% CI: 1.62–10.85) (Appendix 1).

Despite largely correct responses to the survey questions about cervical cancer screening practices, when asked to describe screening in their own words, the majority of men could not provide accurate descriptions.

“*She just said that I went to the hospital, they tested me and they have found that I am okay. So I did not go into detail because I was just happy my wife was okay.”* (50–54 years old, partner screened).

Of those who provided a description of the screening process, most men reported knowing that it includes a vaginal exam; and some men – especially those whose partners had previously been screened and with higher GEM scores – knew that screening is conducted with an instrument or machine (sometimes mentioning that it is metal); and some stated that vinegar is involved.

“*I don’t really know about the process, I only heard that the doctors have access to the woman’s private parts and screen inside there.”* (35–39 years old, partner not screened).

“*She just said they have a machine that they insert and start scanning to check if she has the disease or not.”* (45–49 years old, partner screened).

Many men did however note that screening is important because cervical cancer is dangerous (deadly) unless it is caught early – and older men whose partners had been screened were particularly likely to say this. No men said that screening was unimportant.

“*The disease is dangerous because when it is detected early it is treatable, but if discovered at a later stage, it can cause death*.” (55–59 years old, partner screened).

“*If the wife is not screened but has cervical cancer, then it means their family will be affected. Because of that women need to be screened.*” (55–59 years old, partner screened).

Men mentioned numerous benefits related to cervical cancer screening -- primarily, that screening is the only way to detect problems related to cervical cancer, unlike other health conditions which can be assessed using other diagnostic or laboratory tests. This was more often mentioned among men whose partners have previously been screened.

“*The process is good because they are using instruments to see where eyes cannot see, where there is a problem.*” (50–54 years old, partner screened).

“*It is the only procedure to know whether a person has the disease or not.*” (25–29 years old, partner not screened).

After hearing a brief description of screening, men were asked whether they were comfortable with the procedure or with the idea of their partner being screened. No respondent said that he was uncomfortable or that he would be uncomfortable with his female partner getting screened. However, a few men had hesitations about their female partner being screened by a male provider. The most common reason for this concern was general modesty and shyness. This was more typically mentioned by older men and those with below-median knowledge scores.

“*For a man and a woman being in a room, and one person being naked, it becomes embarrassing.*” (40–44 years old, partner not screened).

Some respondents were also concerned that male providers may have or develop sexual feelings toward their partners due to nudity during the screening process. These were mostly men with lower knowledge scores, and included men with partners who had, and had not, been screened.

“*Sometimes a male doctor might perform the process, so they need to be able to restrain their urges as they might be tempted to sleep with the women.*” (40–44 years old, partner screened).

“*I have heard that male doctors have sexual relations with female patients. If men hear that their wives will be undressed and put on an exam table by a male doctor … we know that once a man sees a woman naked they will want to have sexual intercourse with her. Because of that men hesitate to tell their wives to get screened for cervical cancer.*” (50–54 years old, partner not screened).

Most men, however, were comfortable with male providers performing the procedure. Many pointed out that you do not get to choose the gender of your provider for other procedures, and that providers are professionals with a code of conduct. These comments were more common among men whose partners had previously been screened.

“*If I contract an STI, even a female doctor is at liberty to check my private parts in order to help me*.” (40–44 years old, partner screened).

“*Doctors learn confidentiality in their work, and have a responsibility to do their job. It is not like a female doctor is supposed to treat female patients only.”* (45–49 years old, partner screened).

Some men (mostly younger men) were concerned about pain from the procedure or that screening was dangerous. This was reported by men with and without screened partners, and by men with approximately average knowledge scores. Several men felt that such pain is necessary, and compared this to discomfort during an injection.

*“Because the body is soft and the metal is hard, it can injure the sex organ of the woman*” (40–44 years old, partner not screened).

“*At the hospital we also get injections and you prepare to feel pain but what can you do*” (55–59 years old, partner not screened).

It was more common, however, for men to say that they did not believe screening was painful for women – particularly men whose wives had previously been screened, and men with higher knowledge scores. Some men specifically said that the instrument would not be allowed if it were painful; and that providers would not implement a painful procedure. Others noted that their wives had specifically said it was not painful.

“*I think that the doctors are specialists and when they insert the metal they do it in a way that the patient will not feel pain*” (40–44 years old, partner not screened).

*“I heard about the pain from other people but when my wife came back from being screened, she said they inserted an instrument but she did not say anything about the procedure being painful*” (50–54 years old, partner screened).

### Decision-making about screening

In response to questions about household decision making, approximately 30% of all respondents said that he alone makes decisions about his female partner’s health care. Though the association was not significant, 38% of men with an unscreened partner and 26% of men with a screened partner stated that he alone should make decisions about cervical cancer screening and treatment (Table 2).

**Table 2:** Household decision making and gender norms by partner cervical cancer screening status

	Total	Not previously screened	Previously screened	***p***-value
	***n*** = 120	***n*** = 47	***n*** = 73	
**Female partner involved (alone or jointly) in household decisions, n (%)**				
Major household purchases	44 (37%)	13 (28%)	31 (42%)	0.10
Minor household purchases	104 (87%)	42 (89%)	62 (85%)	0.49
Respondent’s healthcare	90 (75%)	34 (72%)	56 (76%)	0.59
Female partner’s healthcare	88 (73%)	33 (70%)	53 (73%)	0.78
**Cervical cancer services**				
Believes female partner should be involved (alone or jointly) in decisions about screening and treatment, n (%)	83 (69%)	29 (62%)	54 (74%)	0.16
GEM score, mean (IQR)^*^	10.2 (8–13)	9.3 (6–12)	10.8 (8–14)	0.02

^*^16 point scale; higher score indicates more progressive gender views

When asked to describe what role men should play in supporting their partner for cervical cancer screening, many (especially those with above-average GEM scores) said that they should provide encouragement.

*“The husband has a very important responsibility because he has the capacity to encourage the woman to get tested more often for cervical cancer.”* (45–49 years old, partner screened).

Some respondents also noted that they should escort their partners to the hospital for screening. These men were mostly young and had partners who had been screened previously.

*“Men need to protect their wives by taking their wives to get screened for cervical cancer.”* (50–54 years old, partner not screened).

Another role for men was in having conversations with their partners around cervical cancer, specifically regarding risk factors, the screening process, and test results. This role was discussed by men with both low and high GEM scores, and was mentioned more often by respondents with higher than average knowledge scores.

*“This should be treated as a family problem and discussed in order to prevent new cases of infection.”* (40–44 years old, partner screened).

*“They have a huge role of explaining to them the dangers of the cervical cancer, and that it is easy to get help from the hospital when the symptoms are detected, and this may protect her from the dangers of it.”* (50–54 years old, partner not screened).

### Gender norms and screening

Men with screened partners exhibited significantly more equitable gender beliefs on average than men with unscreened partners (mean GEM score of 10.8/16 vs. 9.3/16, *p* < 0.05) (Table 3). This score difference was seen largely in the GEM Sex sub-domain, particularly among younger men (under 45 years old) (7.0/10 vs. 5.6/10, *p* < 0.05).

**Table 3:** Association of GEM and knowledge scores and reported partner screening behavior

	aOR	95% CI
GEM score^**1**^	1.11	0.98–1.26
GEM Sex Domain score^**1**^	1.44	1.02–2.02^*^
GEM Violence Domain score^**1**^	1.07	0.65–1.76
Knowledge score^**2**^	0.98	0.79–1.22

^1^Adjusted model includes age and educational attainment (categorical)

^2^Adjusted model includes age, educational attainment (categorical), GEM score, and knowing someone who died or survived of cervical cancer

^*^p < 0.05

The single GEM element most strongly associated with having a screened partner was disagreement with the statement “It is a woman’s responsibility to avoid getting pregnant” (OR = 2.69, 95% CI: 1.22–5.90, *p* < 0.05) (Appendix 3). Other items with a strong and significant association were “Women who carry condoms are ‘cheap’” (OR = 2.18, 95% CI: 1.03–4.62, *p* < 0.05) and “There are times when a woman deserves to be beaten by her partner” (OR 1.98, 95% CI: 1.01–4.96, *p*-value< 0.05).

In a model that included covariates for age and education level, the GEM sex sub-domain score was significantly associated with having a screened partner, with an adjusted OR of 1.44 (95% CI: 1.02–2.02, *p* < 0.05) (Table 3). There was not a significant association between cervical cancer knowledge score and partner screening status in the multiple variable model.

## Discussion

Our study found high awareness of cervical cancer and widespread acknowledgment of the importance of screening among surveyed men, though some expressed specific concerns over the screening process and most believed they should be involved in their partner’s screening decisions. Over 90% of men surveyed had heard of cervical cancer, with 28% of men knowing someone who ever had cervical cancer. The scant literature on men’s knowledge and attitudes about cervical cancer screening has focused on community-based studies; to our knowledge, ours is the first study on this topic conducted at a facility actively promoting and providing cervical cancer screening. This may partly explain why we found greater knowledge than previous studies: less than one-half of men had ever heard of cervical cancer in a focus group study conducted in urban Ghana, and less than one-third had in a community-based survey in a semi-urban setting in South Africa [36, 37]. Similarly, previous studies have found that fewer men knew someone who had cervical cancer, with 10% of survey respondents in a community-based study from rural and urban sites in Swaziland [33], 5% of respondents in a study conducted at HIV clinics in rural and urban areas of Kenya [35], and no respondents among urban men in Ghana reporting a direct contact [37]. We also found higher knowledge of cervical cancer risk factors and of screening services than has been previously reported -- although some important gaps in knowledge remain [27, 28, 30, 35, 38]. For example, men in our study, compared to a 2014 study among mostly HIV-positive men in Nyonga Province, Kenya [35], were more likely to correctly identify that screening involves examining the cervix for changes (90%, versus 57%), that HIV-negative women are also at risk (87% versus 10%), and that women without symptoms should be screened (100% versus 75%). In both our survey and in qualitative interviews, risk factors related to sexual behaviors were the most frequently cited. In addition to reflecting the unique sample population, these findings may be attributable to the particularly high prevalence of cervical cancer in Malawi, and effects of recent information campaigns, scale-up of screening programs, and a general temporal increase in awareness and knowledge, particularly among people living with HIV where education has been more heavily focused. Nonetheless, previously-reported misconceptions persist in our sample, such as believing that vaginal herbs and poor male hygiene contribute to cervical cancer risk [33, 37]. Additionally, although most men correctly identified general concepts of screening and treatment, most were unable to describe the procedure during qualitative interviews, thus reflecting a limited understanding of the details of screening services. We did not, however, find an association between a man’s knowledge about cervical cancer and his partner’s screening history.

Men with more equitable gender views about sex were significantly more likely to have a screened partner, particularly among younger men (< 45 years of age), suggesting that gender dynamics may be key to women’s uptake of screening services. However, equitable gender views regarding violence were not associated with screening. This is consistent with the limited evidence from sub-Saharan Africa on this topic. A study using national survey data from Kenya found that areas with higher levels of gender equity had higher overall cervical cancer screening rates [52]. The same has been found for other types of women’s health services, including contraceptive use [53] and maternal health care (antenatal and delivery care) utilization [54, 55]. It is possible that less gender equitable views could serve as a barrier to screening, particularly if a man discourages screening or denies his female partner permission This is critical because we found that most men (> 90%) believed they should be involved in their partner’s screening and treatment decisions, and 38% believed they should be the final decision-maker for cervical cancer screening – although we did not find an association between screening behavior and household decision-making dynamics. Other studies have found that men are heavily involved in cervical cancer screening decisions: half of men interviewed from both urban and rural sites in Swaziland and 72% surveyed in rural Malawi reported being the final decision-maker for cervical cancer screening [33, 38]; and a previous national study in Lesotho found that women with more involvement in household decisions were more likely to have heard of cervical cancer screening [56]. Previous qualitative research from both urban and rural areas of Kenya indicated that women without spousal permission for screening would be seen as “sneaking around” [29], and that fears of a husband forbidding the wife to be screened (and the marital discord that would ensue) might cause some women to keep their screening history secret [34].

Even though men who expressed concerns about having their partner screened were in the minority, many participants in our study had persistent worries about the screening procedure, particularly relating to male providers performing VIA and concerns that the procedure was painful. These findings are consistent with studies conducted among men elsewhere in Africa [29, 37]; but there is limited information on interventions or implementation strategies to overcome these barriers in this context, e.g. focusing on training more female VIA providers. Although men in other studies expressed concerns about infertility and the required period of abstinence after treatment [29], this was rarely mentioned by respondents in our study. It is important to note that some respondents were not concerned with the gender of VIA providers, so interventions that leverage peer discussions may be particularly successful in shifting group norms.

As one of the first studies conducted among HIV-positive men engaged in care at a facility that offers cervical cancer screening, and thus possessing some exposure to services, our findings have implications for future cervical cancer screening programs. First, our data show that male engagement and male buy-in for cervical cancer screening services is critically important. Future programs should consider tailored strategies to engage men and gain their buy-in in order to reach the maximum number of women possible. Second, additional messaging may be required around subjects that are of concern to men, including professionalism of providers and pain. Despite comparatively high exposure to and knowledge of services in this study sample, these concerns persisted. Finally, programs may benefit from the option of female only providers, particularly in settings where gender dynamics may affect trust in male providers.

This study is among the first to our knowledge to explore the association between women’s cervical cancer screening history and characteristics of their HIV-positive male partners, thereby making an important contribution to our understanding of men’s views of, and roles in, cervical cancer prevention in Malawi and similar settings. However, there were several limitations that should be noted. First, we assessed screening history as reported by the husband, which reflects both true screening behavior and the man’s knowledge of this, which may be influenced by relationship dynamics and communication. Future studies using partner dyad surveys would more accurately measure screening history, and explore the reliability of male partner reporting. Second, this study may suffer from social desirability bias, particularly during qualitative interviews about the acceptability of screening; although we tried to mitigate this bias by using a trained and highly experienced male interviewer. There also may have been reporting bias during the interview portion, if men were “prompted” by the preceding survey questions. Third, we acknowledge limitations in our study that may be due to unexplored nuance in socio-cultural factors. In particular, while the GEM scale has been widely validated and used in multiple African settings [57–62], it has been used minimally in Malawi. Future studies should explicitly address mechanisms through which the local cultural context may affect screening decisions, as well as possible differences by cultural and tribal group. Fourth, models exploring the association between partner screening and male characteristics relied on assumptions about underlying variable relationships and confounders, as delineated by the DAG models. Lastly, this study was conducted in HIV-positive men engaged in ART care at a facility with an active cervical cancer program, a population that may not be representative of most men in Malawi or the region, and who may also have higher-than-average exposure to medically accurate health information and health messaging. The use of convenience sampling of men awaiting ART services may further limit generalizability. Further research should be conducted at more sites and with diverse populations.

## Conclusion

Men play an essential role in decisions about women’s health in settings such as Malawi, yet relatively little research has properly identified and recorded their understanding, beliefs, and concerns about cervical cancer prevention. We found that while HIV-positive men are generally aware and supportive of cervical cancer prevention, several hesitations persist. These may influence women’s uptake of screening, therefore engaging men and addressing men’s concerns will be key to achieving the ambitious global goals outlined in the 2018 WHO Call to Action to eliminate cervical cancer as a public health problem worldwide.

## Supplementary information

**Additional file 1.** Quantitative survey.

**Additional file 2.** Qualitative interview guide.

**Additional file 3.** Directed Acyclic Graphs (DAGs) for multivariate model selection.

**Additional file 4.** Study recruitment, eligibility, and analysis populations.

## Data Availability

The datasets used and/or analysed during the current study are available from the corresponding author on reasonable request.

## Appendix 1

**Table 4:** Experiences and perceptions of cervical cancer by partner screening status

	Total	Not previously screened	Previously screened	p-value
	***n*** = 120	***n*** = 47	***n*** = 73	
**Cervical cancer contacts**				
Knows someone who has survived CC	12 (10%)	4 (9%)	8 (11%)	0.66
Knows someone who has died of CC	25 (21%)	7 (15%)	18 (25%)	0.20
**Perceived danger, relative to HIV**				
CC is equally or less dangerous	28 (23%)	13 (28%)	15 (20%)	0.37
CC is more dangerous	92 (77%)	34 (72%)	58 (79%)	
**Perceived danger, relative to high blood pressure**				
CC is equally or less dangerous	73 (61%)	31 (65%)	42 (57%)	0.36
CC is more dangerous	47 (39%)	16 (34%)	31 (42%)	
**Believes partner could get CC throughout her life**				
Strongly or somewhat agree	106 (88%)	41 (87%)	65 (89%)	0.76
Somewhat or strongly disagree	14 (12%)	6 (13%)	8 (11%)	

## Appendix 2

**Table 5:** Correct response to knowledge questions about cervical cancer risk factors, screening, and treatment and association with partner ever screened for cervical cancer

	% correct	OR†	95% CI
Risk factor knowledge			
Sex without a condom	91%	0.55	0.14–2.20
Poor diet	47%	0.99	0.48–2.06
Lack of male circumcision	93%	4.88	0.58–41.01
Poor male hygiene	8%	2.77	0.56–13.65
Inherited or genetic causes	69%	0.55	0.43–1.26
Multiple sexual partners	100%	–	–
Washing vagina too vigorously	52%	0.59	0.28–1.24
Having > 5 children	45%	1.02	0.49–2.13
Applying herbs to vagina	6%	0.24	0.04–1.27
Screening and treatment knowledge			
Only HIV+ women are at risk	88%	0.75	0.24–2.35
Screening should begin at 30 years old	35%	1.25	0.58–2.72
Screening should occur even if there are no symptoms	100%	–	–
Screening looks for changes on the cervix that indicates a woman is at risk for cancer	91%	1.33	0.38–4.63
Screening takes place at this clinic	80%	4.20	1.62–10.85^******^
Treating first signs prevents cancer from occurring	96%	1.04	0.17–6.45
Treatment affects fertility	52%	0.83	0.40–1.73
Following treatment, women should not have sex for 4 weeks	33%	2.58	1.11–5.98^*****^

†Univariate association between correct response and having a screened primary partner

^*^*p* < 0.05, ^**^*p* < 0.01

## Appendix 3

**Table 6:** GEM individual element frequencies and association with partner screening status

	% who disagree with this statement^1^	OR^2^	95% CI
A woman’s most important role is to take care of household chores and cook	22%	1.38	0.56–3.40
Men need sex more than women do^1^	47%	1.14	0.55–2.38
You do not talk about sex, you just do it^1^	87%	2.23	0.77–6.48
Women who carry condoms are ‘cheap’^1^	50%	2.18	1.03–4.62^*****^
There are times when a woman deserves to be beaten by her partner^2^	70%	1.98	1.01–4.96^*****^
It is a woman’s responsibility to avoid getting pregnant^1^	42%	2.69	1.22–5.90^*****^
A woman should tolerate being beaten by her partner to keep her family together^2^	69%	1.09	0.49–2.40
I would be outraged if my wife asked me to use a condom^1^	84%	1.49	0.56–4.00

^1^Respondents were asked if they “agree”, “partially agree”, or “disagree” with each statement reflecting a harmful gender norm; responses were categorized as “agree” (partial or complete) or “disagree”

^2^Univariate association between disagreement and having a screened primary partner

^*^*p* < 0.05

## Abbreviations

ART: Antiretroviral therapy
CC: Cervical cancer
CI: Confidence interval
DAG: Directed Acyclic Graph
GEM: Gender Equitable Men
IQR: Interquartile range
OR/aOR: Odds ratio/adjusted odds ratio
VIA: Visual inspection with acetic acid
