# Supplementary material for: “A loving man has a very huge responsibility”: A mixed methods study of Malawian men’s knowledge and beliefs about cervical cancer
Source: BMC Public Health. 2020 Oct 2;20:1494. doi: 10.1186/s12889-020-09552-1 (PMC7532091; doi:10.1186/s12889-020-09552-1)
Supplement: Supplementary file 1 — Additional file 1. Quantitative survey. [file 12889_2020_9552_MOESM1_ESM.docx]

|  |  |
| --- | --- |
| **SECTION A: Demographics** |  |
| Q1. What is the highest level you completed in school? | No School |
|  | Primary 1-4 |
|  | Primary 5-8 |
|  | Secondary |
|  | University |
| Q2. How many children under the age of 15 live with you (sleep at least 3 nights a week at the same house)? | [Integer response] |
| Q3. How many adults 15 years or older live with you (sleep at least 3 nights a week at the same house)? | [Integer response] |
| Q4. Do you have one or multiple wives? | One  Multiple  Prefer not to answer |
| Q5. Have you had multiple sexual partners over the past 6 months? | Yes  No  Prefer not to answer |
| Q6. Think about your primary (main) partner. For the rest of this survey, I will ask you questions about this partner. How old is she? | [Integer response] |
| Q7. For how many years have you been living with this partner? | [Integer response] |
| Q8. What is this partner’s HIV status? I will read five responses and at the end you should tell me your response. | Diagnosed positive, on treatment  Diagnosed positive, not on treatment  Recently diagnosed negative  Status unknown  Prefer not to answer |
| *If Diagnosed positive, on treatment:*  Q8b. Does your wife receive ART here at PIH? | Yes  No  Do not know  Prefer not to answer |
| Q9. Please think of the past 12 months, how would you describe your primary occupation? | Wage employment excluding casual work  Household or self-run business, not including agriculture  Household or self-run business, agriculture  Casual work |
|  | Not working  Prefer not to answer |
|  |  |
| Q10. Over the past 12 months, how would you describe your household income?  *Read all responses to participant* | Allowed me to build my savings  Allowed me to save just a little  Only just met my expenses  Was not sufficient, so needed to use savings to meet expenses  Was really not sufficient, so needed to borrow to meet expenses  Other, specify  Prefer not to answer |
| **SECTION B: Awareness**  In this section, I would like to ask you about your knowledge of cervical cancer and cervical cancer prevention services. | |
| Q11. Do you know anyone who has died of cervical cancer?  *If No or Do not know, skip to Q13* | Yes |
|  | No  Do not know |
|  | Prefer not to answer |
| Q12. If yes, what is your relationship with this person? | Wife/partner  Other family member, specify  Friend or acquaintance  Other, specify  Prefer not to answer |
| Q13. Do you know anyone who has cervical cancer and survived?  *If No or Do not know, skip to Q15* | Yes  No  Do not know  Prefer not to answer |
| Q14. If yes, what is your relationship with this person? | Wife/partner  Other family member, specify  Friend or acquaintance  Other, specify  Prefer not to answer |
| Q15. Have you ever discussed cervical cancer with your wife or partner? | Yes  No  Prefer not to answer |
| Q16. Now I shall describe screening for different diseases as a way of preventing illness, then I’ll ask you questions specifically about cervical cancer screening. A screening test is a test that can detect a disease at an early stage, before you even have symptoms, so it can be treated early and never cause a problem. For many things, like cancer, if you screen for them, you can prevent the disease from ever happening. As an example, pregnant women get blood and urine tests done during ANC even if they feel fine – these are screening tests to make sure the mother and baby stay healthy; or, you may have your blood pressure taken, which is also a screening test. | |
| Have you ever heard of cervical cancer screening, which looks for first signs of cervical cancer (visual inspection, VIA*)?*  *If No or Prefer not to answer, skip to Q20* | Yes |
|  | No |
|  | Prefer not to answer |
|  |  |
| Q17. Have you ever discussed cervical cancer screening with your wife or partner? | Yes |
|  | No |
|  | Prefer not to answer |
| Q18. Have you ever heard of treatment for the first signs of cervical cancer (cryotherapy or thermocoagulation)?  *If No or Prefer not to answer, skip to Q20* | Yes |
|  | No |
|  | Prefer not to answer |
|  |  |
| Q19. Have you ever discussed treatment for the first signs of cervical cancer with your wife or partner? | Yes |
|  | No |
|  | Prefer not to answer |
| **Have you heard about cervical cancer screening and treatment from the following?**  *For each, response options*: Yes, No, Prefer not to answer | |
| Q20. Friends |  |
| Q21. Family |  |
| Q22. Radio announcement |  |
| Q23. Public announcement |  |
| Q24. Television |  |
| Q25. Health clinic or hospital |  |
| Q26. Billboard or posters |  |
| Q27. Other | *If yes, Please specify other [Free response]* |
| **SECTION C: Knowledge** |  |
| In this next section I'd like to discuss your understanding of cervical cancer and cervical cancer services. | |
| Q28. Please tell me if you agree with the following statement:  A virus known as human papilloma virus or HPV is the cause of cervical cancer | Agree |
|  | Disagree |
|  | Do not know |
| **Please tell me if you agree or disagree that each of the following is a risk factor for cervical cancer:** | |
| Q29. Having sex without a condom | Agree |
|  | Disagree |
|  | Do not know |
| Q30. A poor diet | Agree |
|  | Disagree |
|  | Do not know |
| Q31. Sex with a male partner who is not circumcised | Agree |
|  | Disagree |
|  | Do not know |
| Q32. Sex with a male partner who practices poor hygiene | Agree  Disagree  Do not know |
| Q33. Inherited or genetic causes | Agree |
|  | Disagree |
|  | Do not know |
| Q34. Sex with somebody who has multiple sexual partners | Agree |
|  | Disagree |
|  | Do not know |
| Q35. Washing the vagina too vigorously | Agree |
|  | Disagree |
|  | Do not know |
| Q36. Having more than 5 children | Agree |
|  | Disagree |
|  | Do not know |
| Q37. Applying herbs inside the vagina | Agree |
|  | Disagree |
|  | Do not know |
| Q38. How confident were you when answering these questions? | Very sure, I knew all the answers  Somewhat sure, I knew some answers and guessed or did not know some answers  Not sure, I guessed or did not know the answers |
| **Please tell me if you agree with the following statements about cervical cancer screening and treatment:** | |
| Q39. Only HIV+ women are at risk of cervical cancer | Agree |
|  | Disagree |
|  | Do not know |
| Q40. Women should begin screening for cervical cancer at 30 years old | Agree |
|  | Disagree |
|  | Do not know |
| Q41. Women should get screened even if they have no symptoms | Agree  Disagree  Do not know |
| Q42. Cervical cancer screening tests look for changes on the cervix that indicates a woman is at risk for cancer | Agree |
|  | Disagree |
|  | Do not know |
| Q43. This facility, Moyo Clinic, screens for cervical cancer | Agree |
|  | Disagree |
|  | Do not know |
| Q44. Treating first signs of cervical cancer stops that cancer from starting | Agree |
|  | Disagree |
|  | Do not know |
| Q45. If a woman is treated for first signs of cervical cancer, it makes it difficult for her to bear children in the future | Agree |
|  | Disagree |
|  | Do not know |
| Q46. After receiving treatment for the first signs of cervical cancer, women must not have sex for 4 weeks | Agree |
|  | Disagree |
|  | Do not know |
| Q47. How confident were you when answering these questions? | Very sure, I knew all the answers  Somewhat sure, I knew some answers and guessed or did not know some answers  Not sure, I guessed or did not know the answers |
| **SECTION D: Past experiences, severity and susceptibility** | |
| Q48. Has your wife or partner ever been screened for cervical cancer?  *If No, Do not know, or Prefer not to answer, skip to Q51* | Yes |
|  | No |
|  | Do not know |
|  | Prefer not to answer |
| Q48b. Was your wife screened for cervical cancer within the past 6 months? | Yes  No  Do not know  Prefer not to answer |
| Q49. Has your wife or partner ever received an abnormal cervical cancer screening result or is at risk of cervical cancer?  *If No, Do not know, or Prefer not to answer, skip to Q51* | Yes |
|  | No |
|  | Do not know |
|  | Prefer not to answer |
| Q50. Has your wife or partner ever been treated for an abnormal cervical cancer screening result? | Yes |
|  | No |
|  | Do not know |
|  | Prefer not to answer |
| Q51. How dangerous would you rate cervical cancer, compared to HIV? | Cervical cancer is less dangerous than HIV  They are about equally as dangerous  Cervical cancer is more dangerous than HIV |
| Q52. How dangerous would you rate cervical cancer, compared to high blood pressure? | Cervical cancer is less dangerous than high blood pressure  They are about equally as dangerous  Cervical cancer is more dangerous than high blood pressure |
| Q53. How strongly do you agree with the following statement:  Throughout her life, my partner could get cervical cancer | Strongly agree |
|  | Somewhat agree |
|  | Somewhat disagree |
|  | Strongly disagree |
| Q54. There are vaccines that can be used to prevent cervical cancer that are becoming available in Malawi. | |
| If this vaccine becomes available to your daughter through a school-based immunization program, how likely would you be to support your daughter to receive this vaccine? |  |
|  | Very unlikely |
|  | Fairly unlikely |
|  | Fairly likely  Very likely |
|  | Do not know |
| **SECTION E: Decision making and norms** | |
| Q55. Who within your family usually makes decisions about making major household purchases? | Me only |
|  | Wife or partner only |
|  | Myself and wife or partner jointly |
|  | Someone else  Prefer not to answer |
| Q56. Who within your family usually makes decisions about making purchases for smaller daily household needs? | Me only |
|  | Wife or partner only |
|  | Myself and wife or partner jointly |
|  | Someone else  Prefer not to answer |
| Q57. Who usually makes decisions about healthcare for yourself? | Me only |
|  | Wife or partner only |
|  | Myself and wife or partner jointly |
|  | Someone else  Prefer not to answer |
| Q58. Who usually makes decisions about healthcare for your wife or partner? | Me only |
|  | Wife or partner only |
|  | Myself and wife or partner jointly |
|  | Someone else  Prefer not to answer |
| Q59. Who within your family should make decisions about whether your wife is screened for cervical cancer? | Me only  Wife or partner only  Myself and wife or partner jointly  Someone else  Prefer not to answer |
| Q60. Who within your family should make decisions about whether your wife is treated for cervical cancer? | Me only  Wife or partner only  Myself and wife or partner jointly  Someone else  Prefer not to answer |
| **How strongly do you agree with the following statements** | |
| Q61. A woman’s most important role is to take care of her home and cook for her family | Agree  Partially agree  Do not agree  Do not know |
| Q62. Men need sex more than women do | Agree  Partially agree  Do not agree  Do not know |
| Q63. You do not talk about sex, you just do it | Agree  Partially agree  Do not agree  Do not know |
| Q64. Women who carry condoms on them are “cheap” | Agree  Partially agree  Do not agree  Do not know |
| Q65. There are times when a woman deserves to be beaten | Agree  Partially agree  Do not agree  Do not know |
| Q66. It is a woman’s responsibility to avoid getting pregnant | Agree  Partially agree  Do not agree  Do not know |
| Q67. A woman should tolerate violence to keep her family together | Agree  Partially agree  Do not agree  Do not know |
| Q68. I would be outraged if my wife asked me to use a condom | Agree  Partially agree  Do not agree  Do not know |
