# Supplementary material for: “A loving man has a very huge responsibility”: A mixed methods study of Malawian men’s knowledge and beliefs about cervical cancer
Source: BMC Public Health. 2020 Oct 2;20:1494. doi: 10.1186/s12889-020-09552-1 (PMC7532091; doi:10.1186/s12889-020-09552-1)
Supplement: Supplementary file 2 — Additional file 2. Qualitative interview guide. [file 12889_2020_9552_MOESM2_ESM.docx]

| **SECTION F: Interview** |  |
| --- | --- |
| Now I am going to ask you some open ended questions about cervical cancer screening programs. I would like for you to speak freely and openly. I would also like to audio record this section; however your name will not be used and audio will be not be linked to you in any way. | |
| I am now going to begin recording. You may choose not to answer any question, and you may ask me to stop recording at any time.  Does participant agree to continue? Yes  No    *If no, skip to Post-Survey Health Education Script*  *If Yes: Interviewer: Turn on the recorder now. State the date and the interview ID number.* | |
| Q69. What do you think is the role of men in the  prevention of cervical cancer for their wife or partner? | [Free response] |
| Q70. Could you please explain in your own words how cervical cancer screening is done? | [Free response] |
| Q71. I will now briefly describe how cervical cancer screening is done:  A woman is placed on an exam table so that a provider can examine her. The provider uses a small instrument to look at the cervix. If everything is okay, she will be told to come back after a couple of years to be screened again. If the provider sees a problem, there is a short procedure to treat the affected area right then.   What makes you comfortable? What makes you uncomfortable or worried?  Overall, would you feel comfortable having your wife screened? | [Free response] |
| *If wife/partner previously treated (Yes to Q48):*  Q72. Earlier, you said that your wife or partner has received cervical cancer screening. Did she face any barriers to receiving this screening or problems with screening? | [Free response] |
| *If wife/partner previously treated and screened (Yes to Q48 and Q50):*  Q73. You said earlier that your wife or partner also received treatment for an abnormal cervical cancer screening result. Did she face any barriers to receiving this treatment or problems with treatment? | [Free response] |
| *If wife/partner had abnormal screening result but was not treated (Yes to Q48, No to Q50):*  Q74. You said earlier that your wife or partner had an abnormal cervical cancer screening result, but did not receive treatment. Why not? | [Free response] |
| Q75. We are looking to develop a program that can get more women screened and tested for cervical cancer. As a male partner, what do you think we can do to get more women screened and treated? | [Free response] |
| Q76. Is there anything else you would like to tell us? | [Free response] |
