# Supplementary material for: “A loving man has a very huge responsibility”: A mixed methods study of Malawian men’s knowledge and beliefs about cervical cancer
Source: BMC Public Health. 2020 Oct 2;20:1494. doi: 10.1186/s12889-020-09552-1 (PMC7532091; doi:10.1186/s12889-020-09552-1)
Supplement: Supplementary file 3 — Additional file 3. Directed Acyclic Graphs (DAGs) for multivariate model selection. [file 12889_2020_9552_MOESM3_ESM.pdf]

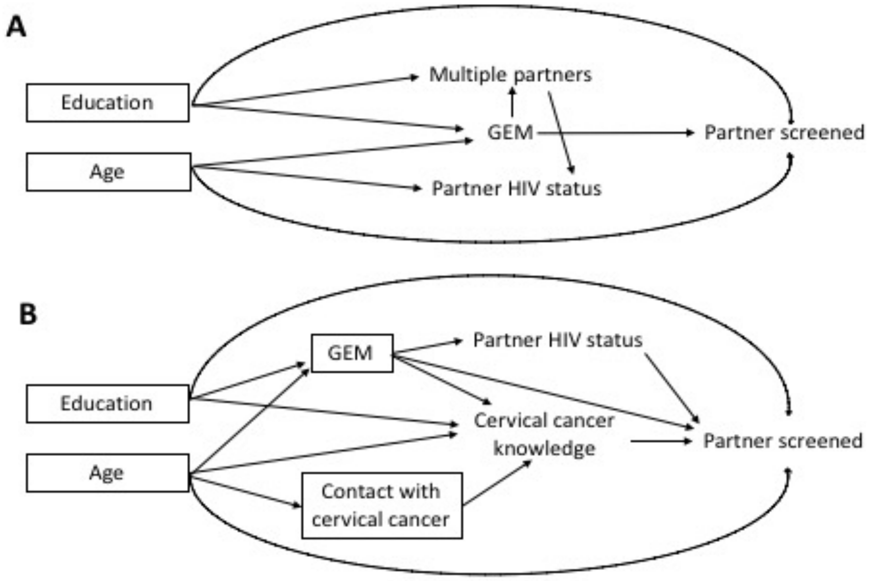

Directed Acyclic Graphs (DAGs) summarizing causal relationships between variables for inclusion as potential confounders in multivariable analyses of the relationship between **A)** GEM and partner screening status, and **B)** cervical cancer knowledge and partner screening status. Variables included as model covariates are enclosed in boxes.
