# Supplementary figures and images for: “A loving man has a very huge responsibility”: A mixed methods study of Malawian men’s knowledge and beliefs about cervical cancer
Source: BMC Public Health. 2020 Oct 2;20:1494. doi: 10.1186/s12889-020-09552-1 (PMC7532091; doi:10.1186/s12889-020-09552-1)

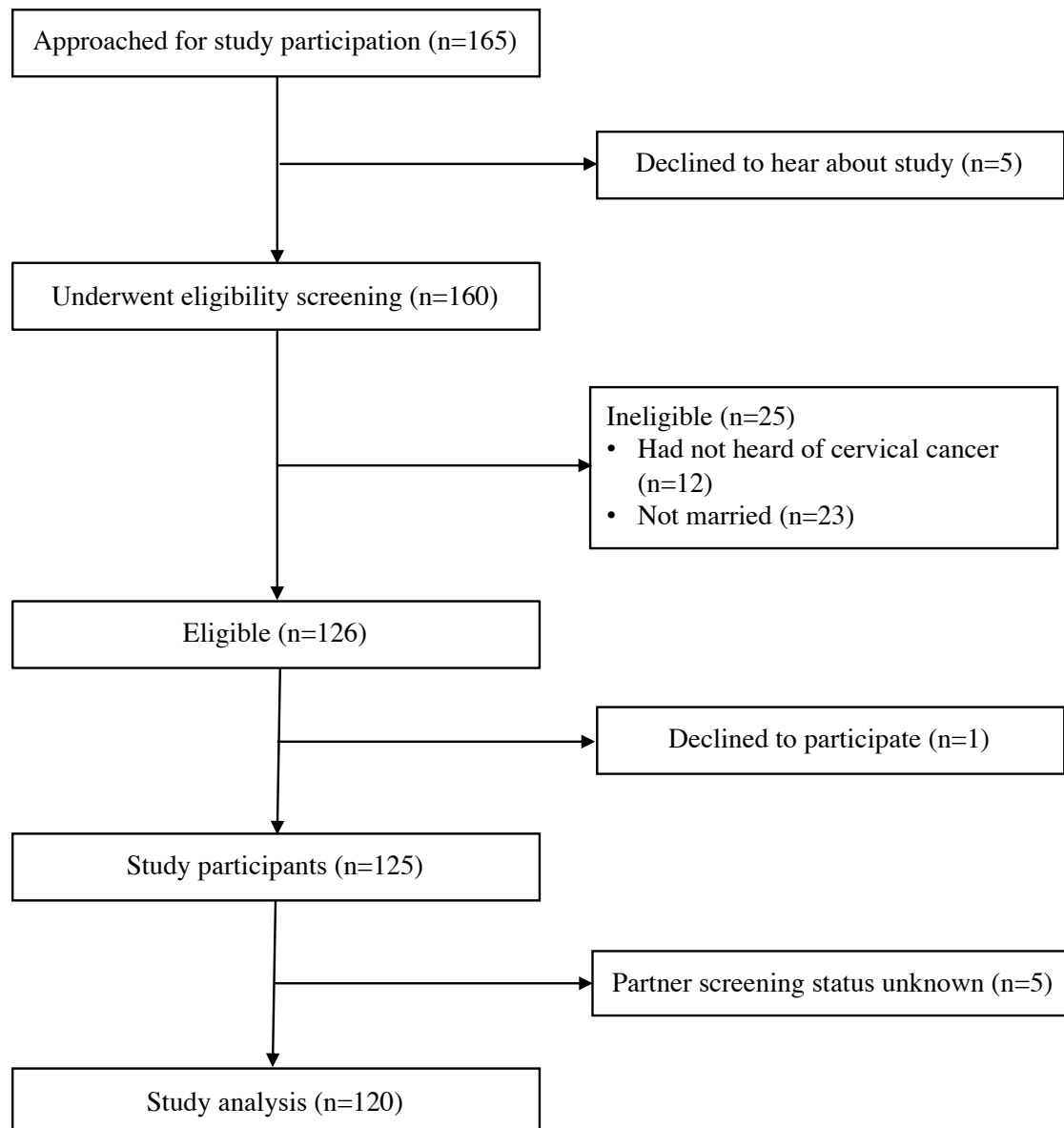

Supplement: Supplementary file 4 — Additional file 4. Study recruitment, eligibility, and analysis populations. [file 12889_2020_9552_MOESM4_ESM.pdf]
